# Student-focused virtual histology education: Do new scenarios and digital technology matter?

**DOI:** 10.15694/mep.2017.000154

**Published:** 2017-09-07

**Authors:** Szabolcs Felszeghy, Sanna Pasonen-Seppänen, Ali Koskela, Anitta Mahonen

**Affiliations:** 1 Institute of Dentistry and Institute of Biomedicine; 2 Institute of Biomedicine; 3 Institute of Medicine

**Keywords:** Webhistology, Student focused education

## Abstract

This article was migrated. The article was marked as recommended.

Innovative changes have become a critical part of teaching when resources are limited. In this study, we examined whether the student-oriented teaching method, when powered by virtual microscopy, improves histology learning compared to traditional microscope-based studies. Anonymous and voluntary post-course surveys were administered to students and essays were processed for content analysis. Google Analytics was used to obtain accurate Internet usage monitoring for WEBMICROSCOPE®. Using SPSS statistics, the examination scores for 2016 were compared to those of previous year, when the course was taught with a traditional-microscope-based model. The results demonstrated that the new teaching scenario was an effective tool, based on the mean examination scores in 2016 compared to the identical groups in 2015. The survey analysis showed that the students benefited more from using WEBMICROSCOPE® and that they frequently gained access to the Web server when they were not in class. The new scenario helped clarify the concept of histology for most of the students and was generally appreciated during teamwork-based histology classes. Students perceived that the use of the digital technology significantly influenced their confidence in learning the fundamentals of histology. In addition, changing to the new teaching scenario powered by WEBMICROSCOPE® improved the students’ motivation to participate in discussions and better understand the concept of Histology between the 2015 and 2016 academic years. Finally, these changes all had a positive impact on the students’ attention and satisfaction.

## Introduction

Together with biochemistry and physiology, anatomy is one of the basic sciences taught in the medical curriculum ( Bergman and Goeij 2010). For many clinical specialties, a long-lasting familiarity with macroscopic and microscopic anatomy is indispensable to guarantee safe and efficient everyday clinical work ( Fasel et al. 2016). Millennium imaging technologies have become an important part of teaching the concepts of human histology and pathology in many medical schools. The emergence of new digital technologies has started to replace traditional information communication in basic science education because the current generation of graduates has been immersed in technology since their early school years and thus have high expectations regarding digital resources. Educators should especially support their proficiency in problem solving in technology-rich environments (Breslauer et al. 2006; Moreno-Walton et al. 2009; Van Nuland et al. 2017; Sharma and Kamal 2006). The traditional microscope-based, teacher-focused program in histology education is very much dependent on having adequate numbers and equal qualities of human histological slides and requires a number of qualified educators who can provide simultaneous close supervision at individual microscope workstations. Currently, the lack of these factors has driven many medical and dental faculties towards Web-based histology education. In the process, Web-based education is an effective way of interactively teaching younger generations. Moreover, by using virtual slides and Internet-based software, students can better understand the concept of histology, especially when using a high-quality digital slide of the tissue of interest.

The internationally highly recognized tradition of providing classical and didactic teaching impetuses, instruction and guidance regarding medical/dental education started at the Kuopio campus in 1972, when our first anatomy educators decided to provide a stimulating intellectual environment for undergraduate students. As one of the flagship institutions in Finland, the Institute of Biomedicine`s mission for education is still to emphasize programs with a strong correlation between structure and function based on Anatomy, Biochemistry, Histology and Physiology. However, there is now a strong tendency for traditional classroom lessons to share a smaller proportion of contact hours between our medical and dental curricula.

**Figure 1.**
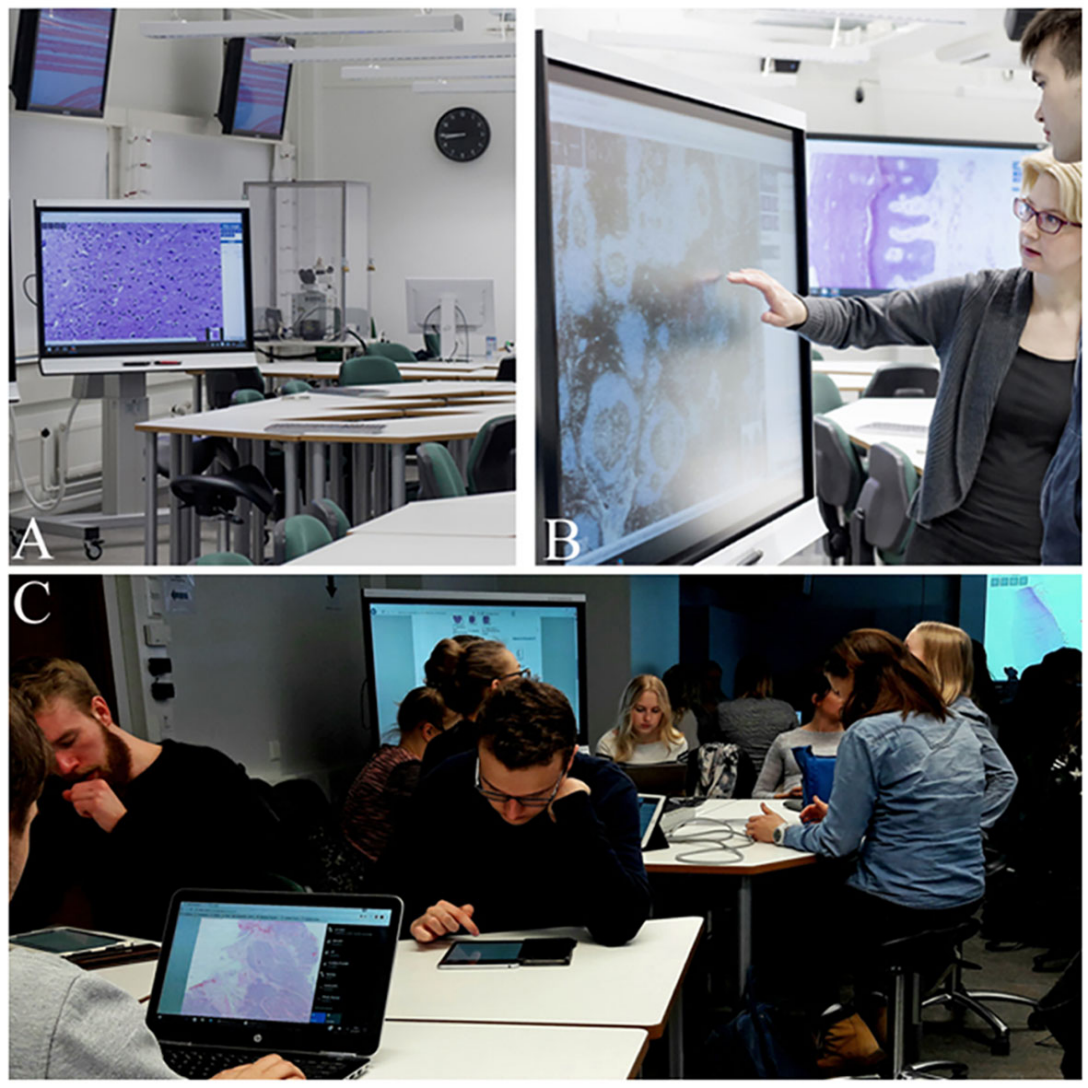
The new profile of the histology classroom at UEF.

It was a significant challenge for the educators at the Institute of Biomedicine to adopt a new more effective and thought-provoking teaching program, particularly in histology, for medical and dental students during the 2016 academic year. Several Web-based microscope applications are currently available, and some of the advanced ones are listed here: (a) NYU Virtual Microscope (NYUVM) from New York University, USA ( https://virtualmicroscope.iime.cloud/), (b) VSlides (Pathorama) from Basel University, Switzerland ( http://pathorama.ch/vslides/), (c) vMic from Basel University, Switzerland ( https://histodb11.usz.ch/index.html), (d) WEBMICROSCOPE ^®^ (Fimmic, Helsinki, Finland ( http://demo.WR.net/) and (e) 3DHISTECH, Budapest, Hungary ( http://www.3dhistech.com/). During the selection process, we looked at technologies from the perspectives of both students and educators and considered the overall effectiveness of their use in terms of practice. Finally, WEBMICROSCOPE ^®^ combined with a student-focused histology program was adopted for the new histology curriculum at UEF ( Figure 1).

The main aim of these changes was to generate active interaction to exchange knowledge about the subject during histology classes in an intellectual environment supervised by teachers that could encourage a sense of inquiry. However, our hidden aim was that students would continue their web-based self-studies during small team discussions outside class hours using the collection of our digitalized histological specimens.

There are plenty of data available that show that students’ interest in anatomy/histology/pathology can be evoked by incorporating digital imaging and that online access to histological specimens might improve their understanding of the cellular arrangement of human tissues/structures and their complex relationships during health and disease ( Pantanowitz et al. 2012; Foster 2010; Kish et al. 2013; Gu and Ogilvie 2005). However, to the best of our knowledge, there is no such data analysis available on the effects of the changes obtained during the shift from traditional microscope-individual slide based and teacher-focused education to student-focused digital histology education.

Using deidentified data from final written histology exam grades, network profile data and an anonymous-voluntary survey given to students, we report the results of our analysis on the effectiveness of the new histology teaching methods. We also present solid evidence that the overall feedback from the students on student-focused digital histology was highly positive. Considering all of our results and the limitations of this study, we are able to recommend that web-based and student-focused teaching methods be included in histology curricula in the field of health sciences.

## Material and methods

### Analysis of academic performance

**Figure 2.**
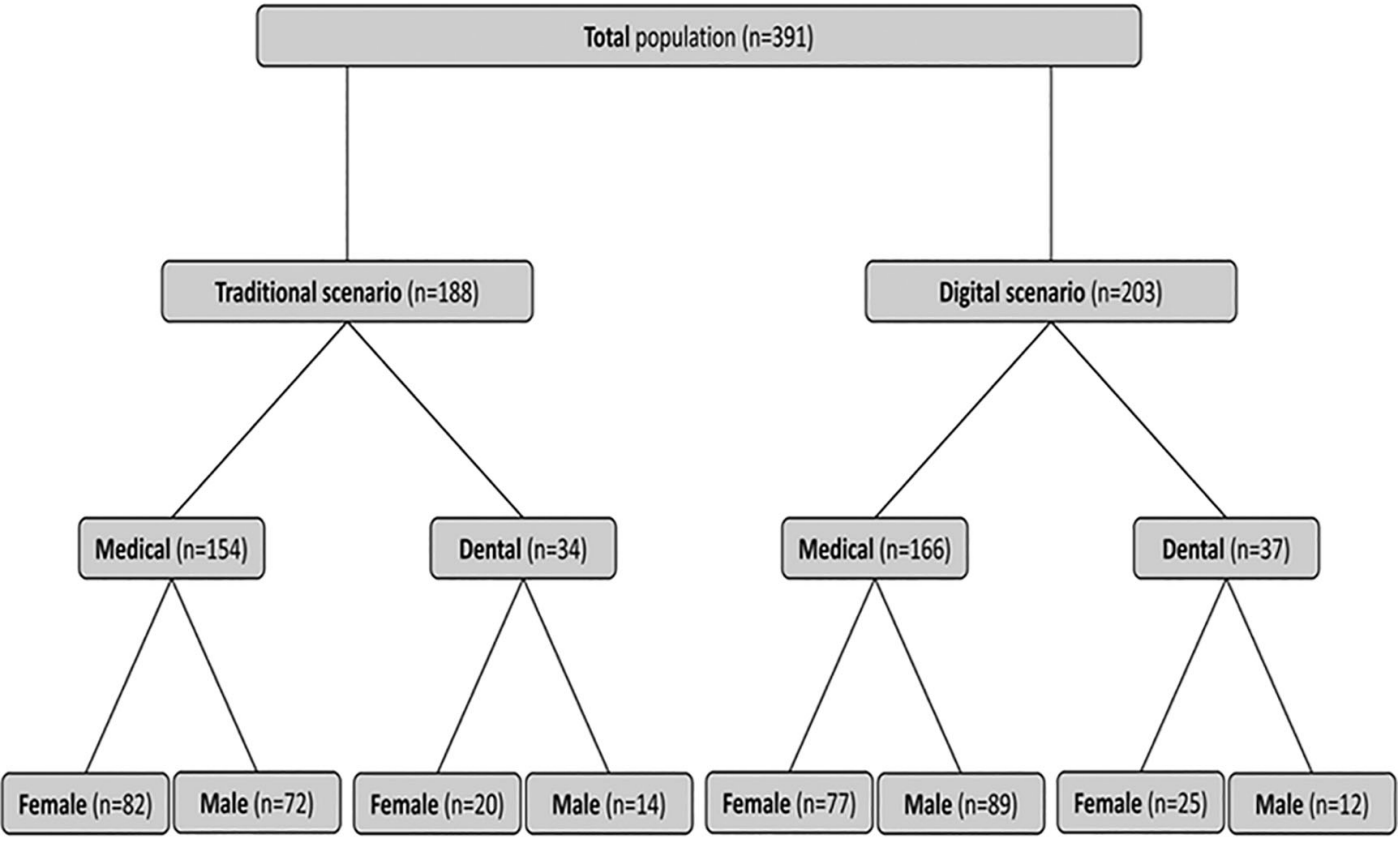
Profile of students participated in the study

We analyzed the core histology written examination performance of 320 medical and 71 dental students in both scenarios (traditional teacher-focused microscope-based/traditional and student-focused WEBMICROSCOPE ^®^-based/digital) separately and together. The total population and gender balance in the medical and dental programs for the two years analyzed are shown in Figure 2. The assessment of histological knowledge was based on the scales and grades on the final subject examinations through the Institute’s data system. Throughout the analysis, deidentified data were used. Only gender information was merged with the individual scores, so no student privacy issues were considered. Therefore, the study protocol did not require submission to the University Ethics Committee. Data are presented as the mean ± standard error of the mean (SEM) when comparing grades and as percentages when comparing proportions of the highest and/or lowest grades. Data were statistically analyzed using the SPSS software version 23 (IBM Corporation, New York, USA). The data were subjected to Student’s t-test (grade averages) and Pearson Chi-Square analysis (grade proportions). p values < 0.05 were considered statistically significant.

Moreover, Google Analytics provided accurate Internet usage monitoring of the WEBMICROSCOPE ^®^ server to understand how the students behaved in “digital scenarios” after they opened the website to drive better performance during histology classes and/or outside the UEF EDUROAM network. Finally, surveys were administered to the 137 students regarding the merits and impact of using WEBMICROSCOPE ^®^ and the reformed histology teaching experiences on student’s own preparedness. The descriptive, voluntary, anonymous, post-course survey was conducted in early spring 2017 and powered by a Web-based Kahoot application. The questions in the survey addressed the methods of teaching, quality of teaching, teaching tools (WEBMICROSCOPE ^®^) and mode of assessment of students in relation to the histology curriculum. In the survey, students were mainly asked to agree, disagree, or give definitive opinions about the questions raised. Profiles of students who participated in the survey are summarized in table 1.

### CONTEXT

#### Learning environments

Professionals participating in histology education during 2015 and 2016, particularly those delivering histology classroom lectures, were the same in both semesters. The major topics for lectures and classrooms were similar and delivered by the same individuals. The number, length and schedules of histology classrooms were identical in both years. All information is available on the UEF website.

#### Real (2015) histological slides and the establishment of the virtual slide collection (2016)

Until 2015, at the Institute of Biotechnology, light microscopes and sets of real slides were used to view sections of interest and learn the concept of histology in the classroom. The quality of slides was appropriate, but there was some discrepancy between different sets of slide boxes from the nature of histological slide preparations.

In early 2016, when the system was changed to virtual Histology, the most representative digital set of teaching slides was created as follows: first, several microscope slides of each tissue of interest were evaluated by professionals, and only the highest-quality and most representative copy of each tissue of interest was selected for digitizing. After this time- and work-consuming selection procedure, slides were sent to Fimmic (Fimmic Oy, Helsinki, Finland) for digitalization. The digital images were transferred to the server, and a Fimmic-powered WEBMICROSCOPE was used in the histology classroom for the academic year of 2016. After the slides were digitalized and transferred to the server, students were able to access these histological sections at any time.

#### Learning scenario differences between academic semesters 2015 and 2016

During teacher-focused histology education in 2015, when students worked under the guidance of a professor using their own box of slides, no real interactions were initiated by students to better understand the concept of histology. In 2015, a teacher explained and showed the histological structures of the tissue; thereafter, students tried to find the same structures in their own slides by using traditional light microscopes in histology classroom while following the syllabus. The interactions among the students and between the students and the teacher was rather limited when teacher-focused histology education was utilized.

In the student-oriented classrooms used in 2016, students were able to receive the professor’s help if it was needed; however, they worked freely and in an individual or collective way throughout the classes. In these student-oriented classrooms, students studied histological samples in groups ( *team-based learning*) using WEBMICROSCOPE ^®^ on big touchscreens ( Figure 1) and following the same syllabus used in 2015. In these classrooms, the level of interaction was greatly elevated, which probably enhanced the students’ motivation to study histology.

In both years, the syllabus was not a minimum-level summary; instead, its content expected medical and dental graduates to better understand the concept of particular tissues of histology. Furthermore, it has some relevant and immediate applications to their future careers as clinicians and can evoke students’ interest in the importance of knowing the principles of histology.

#### Academic performance monitoring

Learning was measured using an objective written test (exam) to assess the acquisition of histology topics in each of the groups studied. The quality, quantity and type of the questions on the final exam were comparable in these two years analyzed. Both final written tests contained a combination of simple, multiple-choice, and “bell ringer” identification-type questions. The highest possible score in both years was 50 points, and the grading system was identical. Moreover, these examinations were developed by the same professors.

## Results

### A shift from traditional and teacher-focused scenarios to digital and student-focused scenarios improved academic performance.

**Figure 3.**
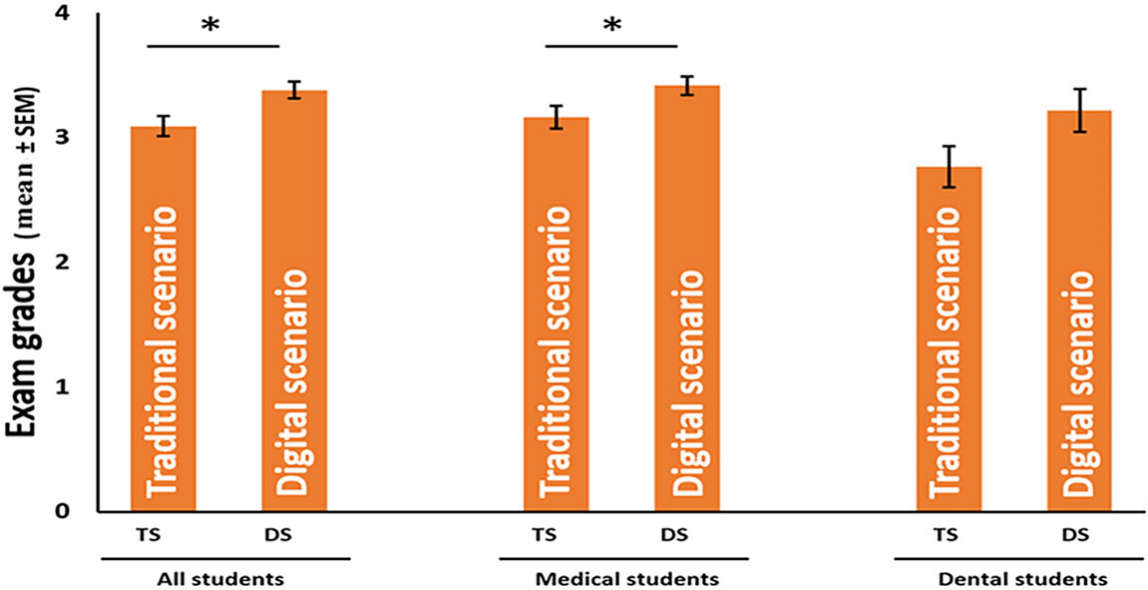
Mean grades of 1st year medical and dental students’ histology exams in traditional and digital scenarios. The data are shown as the mean ± SEM, * p≤0.05, Student’s t-test.

**Figure 4.**
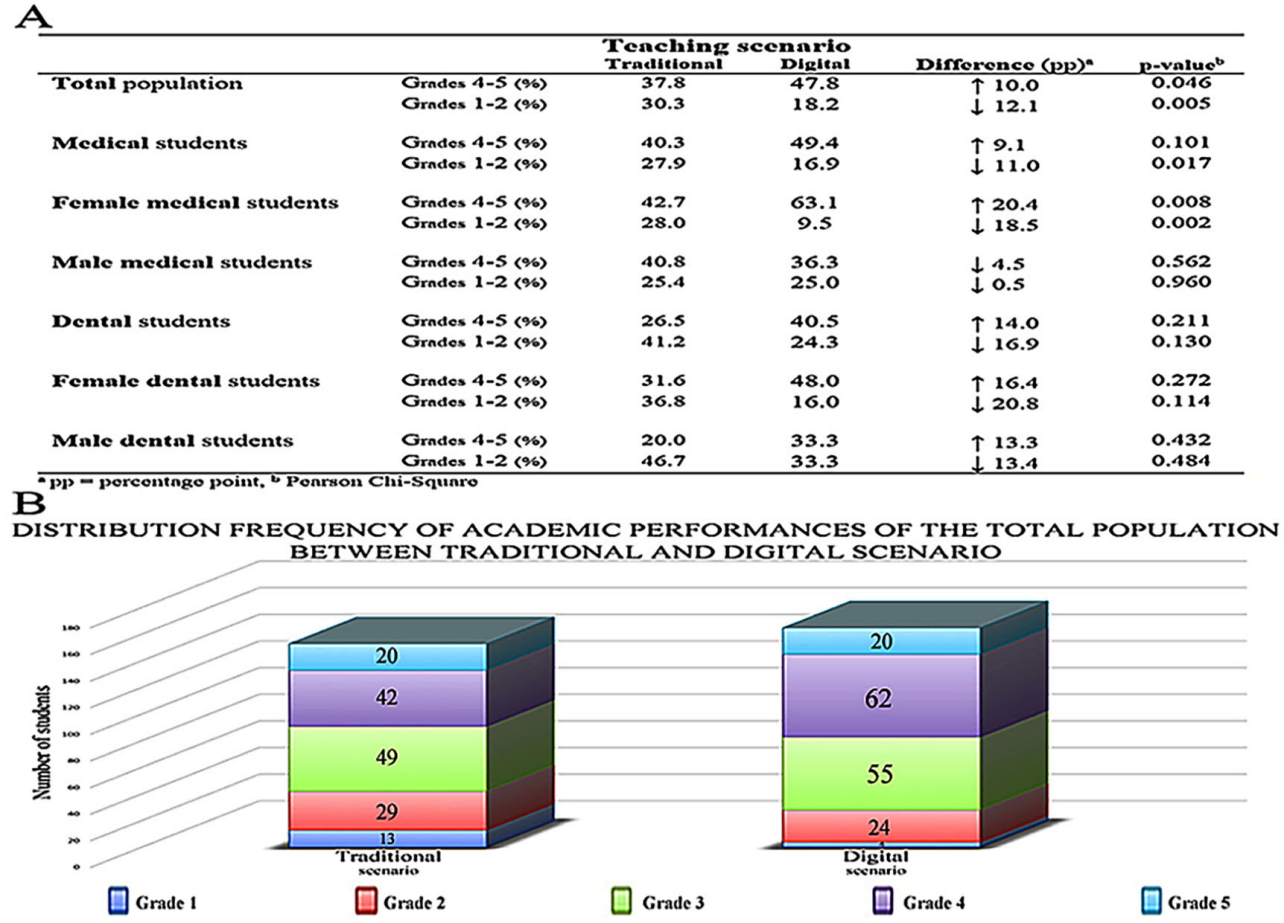
Distribution frequency of academic performances of the different student populations between Traditional and Digital scenarios.

Based on the significantly better performance seen in traditional and digital scenarios ( Figure 3), we decided to analyze further the academic performance of students by comparing the distribution frequencies of the highest (4-5) and lowest (1-2) grades between scenarios ( Figure 4A). The proportion of the highest grades increased significantly in the total population between the traditional and digital scenarios. Moreover, the proportion of the lowest grades decreased significantly, in favor of the digital scenario. Subclass analysis revealed a significant decrease in the proportion of the lowest grades among medical students. However, the proportion of the highest grades did not differ significantly between the scenarios. The proportion of the highest and lowest grades among dental students was not significant, although the trend was similar compared to both the total population and medical students, with a 14.0 percentage point increase in the highest grades and a 16.9 percentage point decrease in the lowest grades, due to the low number of students in each group studied.

### Gender differences

**Figure 5.**
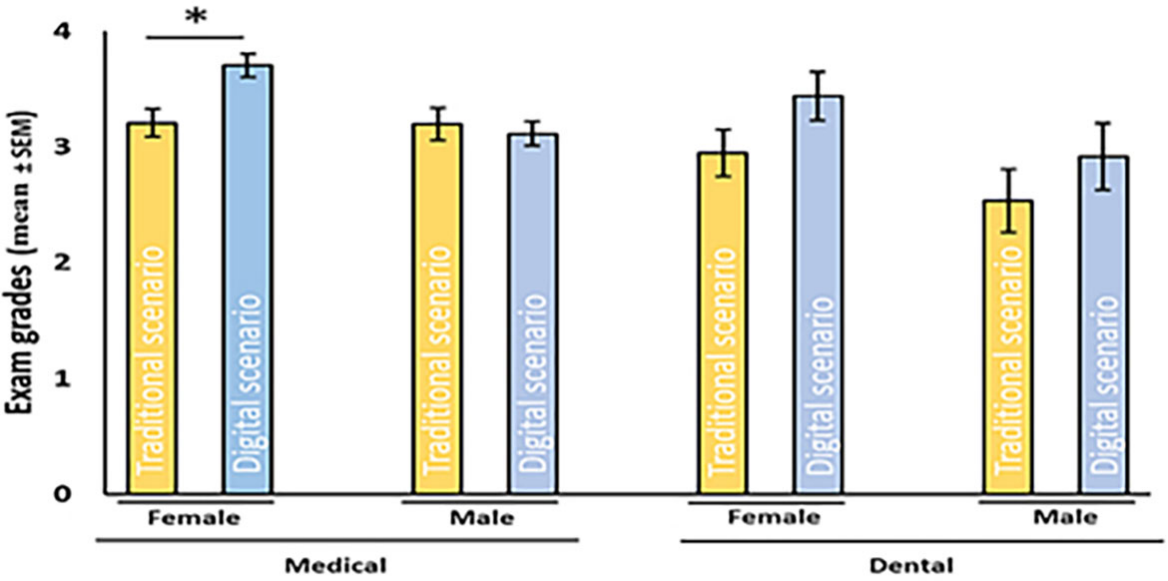
Mean grades of female and male student exams in traditional and digital scenarios. The data are shown as the mean ± SEM, * p≤0.05, Student’s t-test.

The benefit of a digital scenario was significant among female medical students, and there was a similar trend in the female dental student population ( Figure 5). The distribution frequencies of the highest and lowest grades were significantly improved in female medical students ( Figure 4A). Male students did not benefit from the digital scenario, although male dental students performed better in digital scenarios in terms of the average grades and distribution frequencies. However, the effect was not statistically significant. Overall, females seemed to benefit from the digital scenario more than male students did.

**Figure 6.**
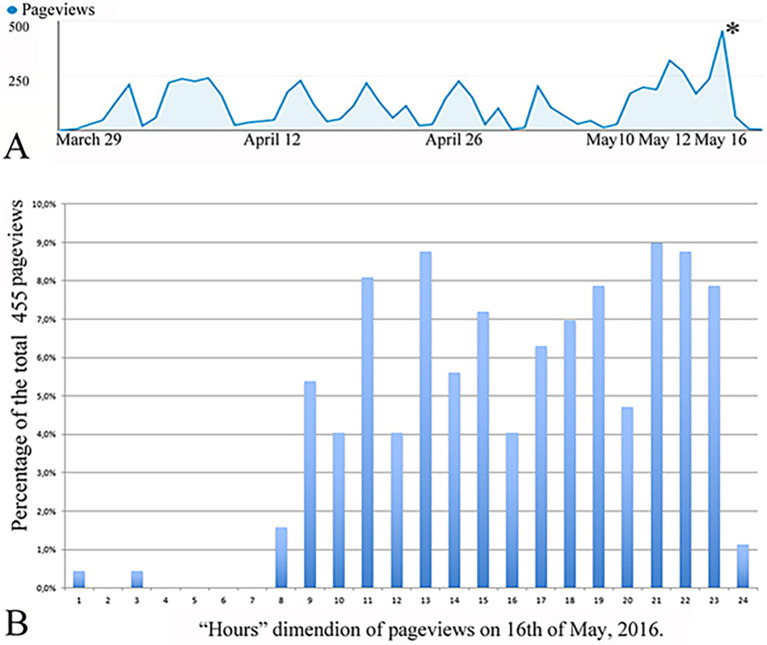
Cumulative graphs of the web browsing activity of the students during the histology course.

Digital and student-oriented scenarios improved students’ academic performance significantly. The next step was to analyze WEBMICROSCOPE ^®^ usage by using Google Analytics and a SEBPQ survey to study the phenomena and reveal possible correlations and explanations. The analysis of the representative survey indicated that 35% of the students with higher grades (4, 5) used the WEBMICROSCOPE ^®^ for up to 4 hours daily while preparing for final written examinations. Further, 10% of this group of students used WEBMICROSCOPE ^®^ daily for more than 4 hours before the histology written exam to increase their knowledge and enhance their self-confidence on histological structure recognition. On the contrary, 42% of those students who passed the exams with the lowest grade used WEBMICROSCOPE ^®^ only up to a maximum of 2 hours daily. According to the survey, it is also clear that the overall time spent on WEBMICROSCOPE ^®^ server sites resulted in more accurate performance on the final written histology examination. These data indicate that students who spend more time in “digital scenarios” are more successful on final written exams. These anonymous, voluntary and objective data given by students are nicely in line with the Google Analytics readouts, which show that just before the final written examination, the “Website visiting activity / page views” peaked around 12 - 16 May 2016 ( Figure 6); further, the total page views during this “high season” numbered well over 1100.

**Figure 7.**
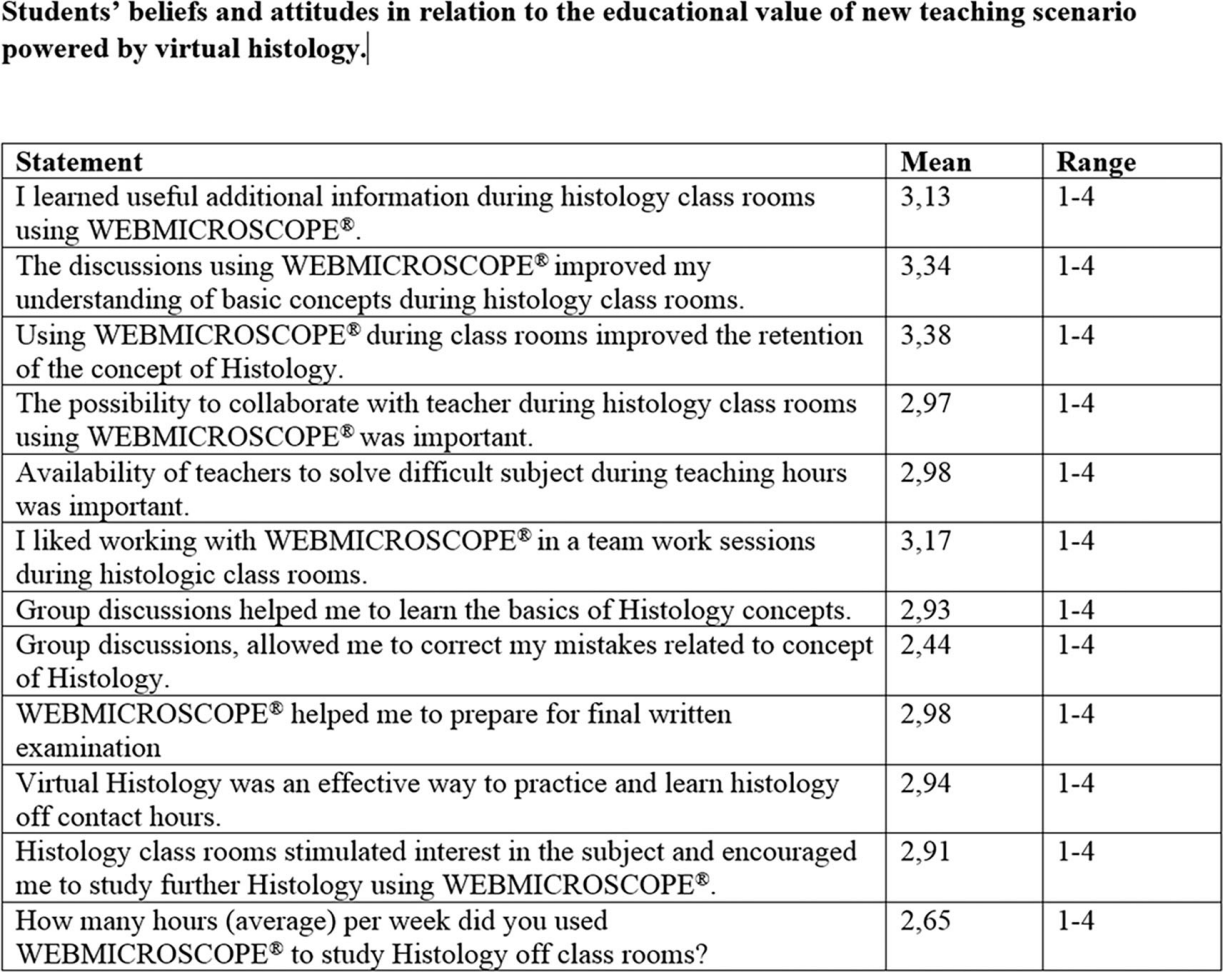
Students’ beliefs and attitudes in relation to the educational value of new teaching scenarios powered by virtual histology.

Next, using the survey, we decided to look beyond the volume-based activity data obtained during the course because we wanted to obtain data about the effectiveness of our renewed histology teaching method: how did students work together during teacher-supervised, student-focused histology classes using their tablets/computers and/or classroom touchscreens running WEBMICROSCOPE ^®^? Were they interested in generating interactions to study at UEF EDUROAM or in public networks? To examine the utilization of WEBMICROSCOPE ^®^, we first used Google Analytics and surveys to answer the questions raised above. Google Analytics provided exact activity reports and information regarding where the students had utilized the WEBMICROSCOPE ^®^ (IP addresses) and indicating whether the students preferred to access the histology slides on or off the UEF EDUROAM network. Furthermore, we obtained valuable answers in the survey concerning the shift from teacher-focused patterns to more student-focused methods, particularly how it influenced the students’ feelings about and understanding of the concept of histology. Finally, we also monitored the effect of regularity of the WEBMICROSCOPE ^®^ site’s utilization on learning for exams in general. First, it is clear from the Google Analytics data that 42% of the total Website hits (6611) were from UEF EDUROAM sites and the rest were from different networks. It is also clear from our survey analysis that students were actively using WEBMICROSCOPE ^®^ during their histology sessions (99%) and initiating small teamwork (97%) problem-focused discussions. Furthermore, students appreciated the supervisors’ WEB-based instructions, which, according to the 98% of the survey answers, helped them understand the basic concept of histology. The survey answers are summarized in Figure 7.

A review of data from surveys ( Figure 7) indicates that students appreciate expert-guided classes on the subject of histology, which helped them in exercises concerning the analysis, synthesis and conceptualization of the fundamentals of histology. Students appreciated the possibility of managing information and using the educator’s reasoning to solve problems. Survey data also revealed that active discussions between students were important to allow them to exchange and exceed their current levels of understanding about histology. According to survey answers, WEBMICROSCOPE ^®^-powered digital histology supervised by an identified expert was a mutually supportive scenario for the students’ confidence: 97% of students had positive perceptions of team work during histology classes. These data of the survey run nicely in parallel with the overall improvement in students’ academic performance and the educators’ individual experiences: teaching the fundamentals of histology may benefit from a shift from teacher-focused/traditional scenarios to digitally directed, WEBMICROSCOPE ^®^-powered, student-oriented education.

It is also important to highlight that the Google Analytics data show that students outside the UEF EDUROAM network intensively logged into the portal to access WEBMICROSCOPE ^®^ from many different 3/4 G networks (mainly from dnainternet.fi, inet.fi, elisa-mobile.fi) and used various web browsers (e.g., Internet Explorer, Safari, Chrome, Edge and Firefox). Additionally, and importantly, survey analysis demonstrated that no loss of functionality was recognized by students using WEBMICROSCOPE ^®^ both in and outside classes and either in or outside the UEF network. According to the survey, more than 78% of the students gained easy access to the server independent of the network profile (3G/4G/EDUROAM) while using different types of devices (tablets in 49% of the cases, desktops in 41%, and smartphones in 9%).

These data suggest that students in younger generations have a favorable impression of having active access to WEBMICROSCOPE ^®^ from outside the UEF network and of surfing uncomplicatedly in the cloud between organized microscopic slides resources.

### Limitations

Because our implementation is rather recent, we have analyzed only the interim semesters of 2015 and 2016. Second, it is important to highlight that the academic quality of the students who experienced the recent change in the histology curriculum may also account for aspects of these successes. However, a pilot experiment regarding this second issue indicated that in the same population of medical students, no improvement could be determined in the anatomy exam grades obtained in 2015 and 2016. Even more interestingly, the average anatomy performance in 2015 was higher than that obtained a year later (2015 mean grade: 3.32; SD: 1.09; 2016 mean grade: 3.26; p<0.05, Student’s t-test). This might decrease the possibility of the issue highlighted above; however, it would be worth the effort to monitor such factors in this issue more accurately in future studies.

## Discussion

In the current educational case report, we uniquely provide empirical evidence that supports the notion that the curricular innovations adopted in 2016 (Institute of Biomedicine, UEF, Finland), which combine digital web “cloud”-stored online histology imaging and student education, are feasible for dedicated students for the following reasons: **1,** educational principles can have a strong impact on students’ academic performance and feelings of competence with regard to learning the fundamentals of histology. **2,** Students easily and actively gained access to new digital tools to analyze histological specimens. **3,** Our education combined independent, outside-class preparations with in-class, small-group, problem-focused discussions. **4,** Web-stored, high-quality and occasionally animated histological slides were available for review. **5,** WEBMICROSCOPE ^®^ allowed both educators and students to “consume” histological images and develop their ideas either during class or via external, web-based discussions. **6,** This “WAW” effect of our new cloud-based histology education motivated us to build a digital bridge in our education for students, and such digital technologies are shown to be pivotal for continuing anatomy, biochemistry and physiology education reforms, thus providing further challenges and motivating opportunities for basic science educators at UEF.

Although free Internet atlases of static histological images are important in terms of becoming familiar with the concept of microscopic anatomy, to teach histology/histopathology, interactive cloud-based “Webmicroscope” servers started to be used only 5-10 years ago (Brisbourne et al. 202; Lundin et al. 2004; Scoville et al. 2007; Pinder et al. 2008; Husmann et al. 2009; Dee 2009; Gona et al. 2012; Sung et al. 2015). Following this international trend in histology education, we changed our classical, static-glass-slide-based and teacher-focused style of sharing information in 2016 to a new IT- and cloud-based system using WEBMICROSCOPE ^®^ software combined with student-focused teaching methods. The aim of these changes was first to bring digital innovations into our education for younger generations and, second, to more efficiently achieve the goal of making students better understand the basic concepts of histology.

In light of the survey seen above, it is important to assert that those students for whom the new histology program was introduced overwhelmingly felt that the new teaching and learning digital scenario had a positive impact on their retention, although there was a “common knowledge” and “consensus” about the very short time limit (10-15 min) of student’s attention during contact hours ( Bradbury 2016).

Of the four studied groups of students (male medical students, female medical students, male dental students and female dental students), only one group (female medical students) showed a significant improvement in academic performance after the switch to student-focused, virtual histology ( Figures 4 and 5) from a statistical point of view. Nevertheless, it is important to highlight that groups of dental students also achieved better exam scores, but based on the small individual number of scores in the group of dental students, the p values were not under 0,05, even though this difference was remarkable in the score distribution patterns. Here, we acknowledge this and attempt to hypothesize about what was special about female medical students. First, it was possible to learn, from the survey data, the differences between the male and female students on how they benefited from this type of learning. Therefore, in practice, digital guidance with digital materials was appreciated more by the girls. This is in line with the data published by Wang and coworkers (2009), who investigated the determinants and age and gender differences in the acceptance of mobile learning, finding that “self-management of learning was stronger determinant of intention for women than for men”. Taking these facts together, it is possible that female students may simply have studied more based on inspiration by the system than they did in the previous traditional microscope-based course, resulting in their better academic performances in 2016.

Ultimately, after analyzing the data, we argued that the investment of time and effort in the design of a new digital and student-centered histology course resulted in deeper and more self-confident learning in digital scenarios for the majority of students from the millennial generation. It is widely accepted that the present generation in universities should be even more characterized as a technologically proficient population than earlier generations and that they are very comfortable with using digital interactive technologies for communication. The use of PCs, tablets and smartphones has become an integral part of learning and everyday life for students ( Kirschner and Karpinski 2010; Hills et al. 2016); therefore, learners might need more creative, interactive and computer-based Internet linked teaching scenarios, even more so than students from earlier generations.

This study offers credible evidence that our educators could deliver the fine details of cellular and tissue organization of human bodies in a more profound way than ever before by using WEBMICROSCOPE ^®^, which can have a huge impact on students’ learning and can help them better understand the fundamentals of histology. In the new digital scenario, students were able to spend more time on learning histology, which definitely increased their learning satisfaction and academic performance.

According to the students’ confidence in teamwork-based discussions obtained from the survey, we believe that studying in peer groups not only appeared to work for better academic performances from basic science but could also provide a foundation for introducing professionalism and leadership skills early in medical education. Teamwork skills in everyday practice are essential in modern healthcare systems that require clinicians to be members of a team that frequently needs to communicate and work together ( O’Connell and Pascoe 2004). Therefore, our new teaching scenario might have a positive impact on this aspect.

Although cell-phone-based platforms have experienced significant improvements since their introduction ( Price 2015; Ingraham 2015) and the younger generation is one of the major users of SMART mobile technology, the Google Analytics data showed that mobiles were used only in 9% of their total server accesses. Currently, SMARTs are nonbulky, highly portable, midrange handhelds with AMOLED (active-matrix organic light-emitting diode) screens, which are better suited to color reproduction and greater viewing angles. This could be optimal for the WEBMICROSCOPE ^®^ server, using its high-quality images (Fleming et al. 2016; Sanchez-Franco 2010). Although larger smartphone screens will lead to a greater perceived control of the subjects ( Kim and Sundar 2014), bigger is not always convenient for use in students’ everyday lives, which might explain the phenomenon noted above. Tablets and desktops (91% of the total accesses from outside the UEF EDUROAM network) might appeal to both task- and affect-oriented needs when studying histology.

To the best of our knowledge, no comparable studies have been published that could be cited in connection with these results. Therefore, further study could offer valuable insights for understanding the effects of screen size on smartphone adoption in digital histology studies, and these studies may confirm and extend our findings by investigating the potential moderating effects of different types of handhelds. Most importantly, the results of this study appear to support the hypothesis that educational principles can have a strong impact on students’ academic performance and feelings of competence with regard to learning the fundamentals of histology, which was educators’ and decision makers’ dream when these educational changes were adopted at UEF.

## Figure Legend

New IT learning environments have been set up to move histology education into digital scenarios to help students’ active exchange of histology knowledge.

Figure A shows the basic arrangement of IT facilities, such as big touchscreens and round tables, to offer possibilities for team work. However, high-quality light microscope is connected to the system for teacher-oriented discussions of real sections of interest, if needed. Figure B illustrates how teachers can interact with students on touch-sensitive screens to motivate students in problem-focused discussions, and C shows how students can use PCs and tablets to gain access to WEBMICROSCOPE ^®^.

Prior permission was sought from individuals present in Figure 1 by the authors in accordance with regulatory requirements.

A total of 391 students took the written examinations; 188 did so in traditional scenarios, and 203 did so in the digital scenario. The mean performance was significantly higher (* /p **≤**0.05/) in the total population of students and in the population of the medical students after the introduction of our new histology education compared to their previous performance. Although the dental students performed definitely better in the new scenario compared to their grades in the old scenario, the difference was not statistically significant (p=0.07).

The table in Figure 4A shows the distribution frequencies of grades in the different populations studied. Figure 4B shows the distribution profile of grades of the 391 students who took the written examinations before (188) and after (203) the new teaching scenario was introduced. Please note that the distribution profiles of grades changed from 2015 (traditional scenario) to 2016 (digital scenario).

A total of 159 female medical students took written examinations before (82) and after (77) the new teaching scenario was introduced. The mean performance was significantly higher after the introduction of our new histology education compared to the previous performance (3.2 versus 3.7, *significant difference between groups p≤0.05).

The graph in figure 6A indicates that students were regularly in “Digital histology scenario” during the time of the course using WEBMICROSCOPE®. Students very actively gained access to the program just before the final exam (labeled with *). The web server traffic pattern demonstrated a baseline activity per week. The number of hits increased to well over 400 in the days prior to the exam. As shown in figure 6B, in the highest season (* 16.05.2015) with 455 total hits, the use of Webmicroscope ^®^ grew considerably during the morning and late afternoon hours, when the students were preparing themselves for the final written examination. From 8-16 h, the Webmicroscope ^®^ Web site received more than 48% of the total “hits”. We also recorded that 84 conversions occurred from the UEF network and that 371 views were gained from outside the UEF network by students, thus indicating the importance of this interactive interface for reviewing histology.

The overwhelming majority of students’ answers indicate that team-based and teacher-supervised discussions using WEBMICROSCOPE ^®^ appear to promote students’ understanding and that educators’ help is respected in this context.

**Table 1.**
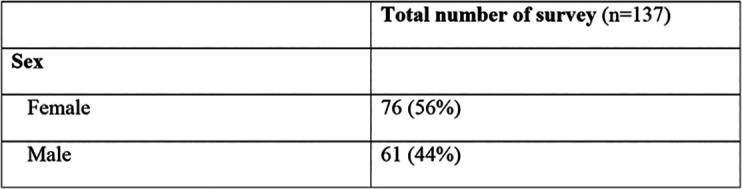
Profile of students that participated in the survey.

Overall, 67.4% of the students completed the survey. Most of the students (>98%) who participated in the surveys had passed a histology final written examination as a qualification to give accurate answers. While it is uncertain how the remaining population of students would have answered the survey questions, we strongly believe that the reported demographics were a fair representation of the class as a whole.

## Take Home Messages

In the University of Eastern Finland, which is one of the flagship universities in Finland and one of the top 300 universities of the world, a new, more effective and evoking teaching program to medical and dental students was adopted in the Institute of Biomedicine during academic year of 2016. According to our data, the student-oriented teaching method, when powered by virtual microscopy, improves histology learning compared to traditional microscope-based studies.

## Notes On Contributors

S. Felszeghy, S. Pasonen-Seppänen designed the study, S. Felszeghy developed, collected survey; S. Felszeghy and A. Koskela analyzed data; S Felszeghy, S. Pasonen-Seppänen interpreted results of experiments; S. Felszeghy wrote and A. Koskela, S. Pasonen-Seppänen edited the manuscript; all authors approved the final version of manuscript submitted for publication.
