# Supplementary material for: Student-focused virtual histology education: Do new scenarios and digital technology matter?
Source: MedEdPublish (2016). 2017 Sep 7;6:154. [Version 1] doi: 10.15694/mep.2017.000154 (PMC10885289; doi:10.15694/mep.2017.000154)
Supplement: Peer review history

# Reviewer report: Reviewer response for version 1

**DOI:** 10.21956/mep.19333.r28234

**Published:** 2018-06-30

**Authors:** Anthony David.M

**Affiliations:** 1 Soho

This review has been migrated. The reviewer awarded 4 stars out of 5 Teaching histology is a complex phenomenon. In the developing world, inadequate resources and incomplete slides compound the situation. This is a refreshingly new method applied. Thank you for the well written paper. The use of latest technology to the hilt is very good. The perceptions of the students are well reflected in the study. The replicability of this depends on resources available but need to be looked into as there is a perceived increase in student learning by this method.

Reviewer Expertise:

NA

No decision status is available

# Reviewer report: Reviewer response for version 1

**DOI:** 10.21956/mep.19333.r28231

**Published:** 2017-10-12

**Authors:** Michael SH Wan

**Affiliations:** 1 University of Notre Dame

This review has been migrated. The reviewer awarded 4 stars out of 5 A well written paper which would help many medical schools in re-designing the delivery of the histology teaching. In the basic science teaching of the medical course, Histology is one of the most poorly attended classes. Students often found the topics really dry and they lack interest in the learning. In the digital era, students are now very tech savvy, the integration of digital learning and feedback would definitely enhance their learning. The detailed description of the new improvement and the monitoring of the students performance in the paper is well presented. The problem based and team teaching are very useful.

Reviewer Expertise:

NA

No decision status is available

# Reviewer report: Reviewer response for version 1

**DOI:** 10.21956/mep.19333.r28233

**Published:** 2017-09-12

**Authors:** Ronald M Harden

**Affiliations:** 1 AMEE

This review has been migrated. The reviewer awarded 4 stars out of 5 A good example of how technology can be used imaginatively to facilitate student centred and collaborative learning. I was not clear how the histology teaching was integrated overall with the students' learning and the extent of the contribution histology should make to the curriculum. None the less, an interesting paper.

Reviewer Expertise:

NA

No decision status is available

# Reviewer report: Reviewer response for version 1

**DOI:** 10.21956/mep.19333.r28236

**Published:** 2017-09-12

**Authors:** Barbara Jennings

**Affiliations:** 1 Norwich Medical School

This review has been migrated. The reviewer awarded 4 stars out of 5 In this article by Felszeghy et al., the successful application of virtual microscopy coupled with a student-centred approach to teaching within the medical school’s Histology curriculum is described, and its rationale is presented in their very useful introduction. I really enjoyed reading the article because:• This type of manuscript (Practical Tips & Guidelines) shares innovative methods that can be considered by the wider community of histology and life sciences teachers and departments• The student focused initiatives allowed for peer-to-peer learning/teaching during classroom time that was supported by faculty, as well as asynchronous virtual learning opportunities. The blended approach fosters self-directed learning.• The authors carefully monitored the curriculum development comparing learning gain and student satisfaction with traditional histology teaching (albeit with test and control cohorts in different academic years). They also measured uptake of virtual learning opportunities. The authors raise some interesting future research questions about determinants-of “self-management” of learning.The article is interesting and well-structured and I will definitely recommend it to other Pathology/Histology/Anatomy teachers.

Reviewer Expertise:

NA

No decision status is available

# Reviewer report: Reviewer response for version 1

**DOI:** 10.21956/mep.19333.r28232

**Published:** 2017-09-08

**Authors:** Trevor Gibbs

**Affiliations:** 1 AMEE

This review has been migrated. The reviewer awarded 4 stars out of 5 Integrating the basic sciences into the medical curriculum, giving relevance to what is taught, introducing new methods of teaching to bring about active learning and keeping up with change are all relevant in todays approach to medical education; hence the relevance of this paper. A well written and informative paper, I really enjoyed reading it and although I felt that the authors concentrated mostly on the change in teaching methodology, I felt it brought in all those other important issues as described above. It was not surprising that the students found the novel use of new technology exciting, they are used to almost living everyday with electronic technology in their hands, but the authors also described the increased interest in a subject that many ( including myself) found rather boring and out of context ( apologies to my histology colleagues) . I didn't put much emphasis on the improvement of assessment scores, given that there are other motivational factors involved hereIn terms of evaluation, it would be interesting to follow this group of students through to later clinical years, although again sometimes the increased motivation to learn can be difficult to measure

Reviewer Expertise:

NA

No decision status is available

# Reviewer report: Reviewer response for version 1

**DOI:** 10.21956/mep.19333.r28235

**Published:** 2017-09-07

**Authors:** Ken Masters

**Affiliations:** 1 Sultan Qaboos University

This review has been migrated. The reviewer awarded 5 stars out of 5 A very well-conducted study, and a very well-written paper. I particularly liked the great care taken by the researchers to ensure that the quality of the material presented was not compromised, and the triangulation of academic results and student perception data. Combining these results with server data adds greater credibility to the researchers’ work.Although the work does occur in a highly-developed and well-supported technological environment, and that may limit the replicability of the study, the researchers have utilised their resources to their full potential. The study underscores the fact that, if technology is to be used in education, it does not merely need to exist, but needs to be used properly and monitored carefully, as the researchers have done.

Reviewer Expertise:

NA

No decision status is available
